# Corrigendum to “Inhibition of HIV-1 replication using the CRISPR/noNLS-Cas9 system as a prophylactic strategy” <[Heliyon 8 (9) (2022) e10483]>

**DOI:** 10.1016/j.heliyon.2022.e12445

**Published:** 2022-12-21

**Authors:** Ali Salimi-Jeda, Maryam Esghaei, Hossein keyvani, Farah Bokharaei-Salim, Ali Teimoori, Asghar Abdoli

**Affiliations:** a Department of Virology, School of Medicine, Iran University of Medical Sciences, Tehran, Iran; b Department of Virology, Faculty of Medicine, Hamadan University of Medical Sciences, Hamadan, Iran; c Department of Hepatitis and AIDS, Pasteur Institute of Iran, Tehran, Iran

In the original published version of this article, in the section entitled “VSV-G-pseudotyped HIV-1 construction”, the authors did not include a part of a sentence they wished to include. “The pseudotyped SCR (single cycle replication) HIV-1 stocks were generated by transfection of HEK-293T cells (7 × 10^5^ cell) with 4 mg of psPAX2 (Addgene plasmid # 12260, Contains HIV-1 gag and pol gene regions) and pMD2.G (Addgene plasmid # 12259, expressing the G protein of the VSV virus) vectors complexed with 20 μl of TurboFect Transfection Reagent in 6-well plate (250 μl plasmid-Turbofect complex in DMEMmedium for each well)” will now be changed to “The pseudotyped SCR (single cycle replication) HIV-1 stocks were generated by transfection of HEK-293T cells (7 × 10^5^ cell) with 4 mg of pNL4-3 (provided by Dr. Kazem Baesi), psPAX2 (Addgene plasmid # 12260, Contains HIV-1 gag and pol gene regions) and pMD2.G (Addgene plasmid # 12259, expressing the G protein of the VSV virus) vectors complexed with 20 μl of TurboFect Transfection Reagent in 6-well plate (250 μl plasmid-Turbofect complex in DMEM medium for each well”.

Also, the authors did not include a sentence in their acknowledgements section that they wished to include. "The authors also would like to thank Dr. Kazem Baesi of the Pasture Institute of Iran for the gift of the VSV-G-pseudotyped HIV-1 vectors used in this project^1^" will now be added to the published article. The authors apologize for the errors. Both the HTML and PDF versions of the article have been updated to correct the errors.

## Footnotes

1 This project was financially supported by grant No 13576 and approved by the ethics committee of Iran University of Medical Sciences (IUMS) with the ethical code IR.IUMS.FMD.REC.1398.137.

